# EFFECT OF CHRONIC RENAL DYSFUNCTION ON THE PERMEABILITY OF THE COLON TO WATER AND ELECTROLYTES: EXPERIMENTAL STUDY IN RATS

**DOI:** 10.1590/0102-672020190001e1472

**Published:** 2019-12-20

**Authors:** Elionai Gomes FREIRE, José Cirlânio Sousa ALBUQUERQUE, Israel Pinto LEAL, Nayara Alves SOUSA, José Ronaldo Vasconcelos da GRAÇA

**Affiliations:** 1 Physiology and Neuroscience Laboratory of the Biotechnology Graduate Program, School of Medicine, Sobral, CE, Brazil; 2 Pharmacology Laboratory of the Federal University of Ceará, Sobral, CE, Brazil

**Keywords:** ENephrectomy, Renal insufficiency, Colon, Water, Electrolytes, Nefrectomia, Insuficiência renal, Cólon, Água, Eletrólitos

## Abstract

**Background:**

Renal insufficiency is a disease that affects several organs by provoking hypervolemia and uremia. The disease reaches more than 500 million people worldwide and few studies bring their influence on the gastrointestinal tract.

**Aim:**

To evaluate the influence of 5/6 nephrectomy-induced hypervolemia on colonic permeability to water and electrolytes.

**Method:**

Sixty male Wistar rats weighing between 280-300 g were divided into three groups: 3, 7 and 14 days after nephrectomy, each one having a false-operated/control and partially nephrectomized. For colonic permeability they were submitted to colonic perfusion with a solution of Tyroad containing phenolphthalein. Differences among the concentrations of Na+, K+ and Cl- were used to calculate the rate of colonic permeability for the electrolytes. Phenolphthalein concentrations were used to evaluate the rate of secretion and water absorption.

**Results:**

The colonic secretion of water and electrolytes occurred expressively in the group seven days after nephrectomy. Hemodynamic and biochemical assessments determined the progression of renal failure in all three groups and polyethylene glycol was shown to be effective in reversing the secretory capacity of the colon.

**Conclusion:**

Hypervolemia established after 7 days post-nephrectomy 5/6 caused marked colonic secretion for water and electrolytes. The organism presents progressive colonic secretion as the blood volume increases; on the other hand, polyethylene glycol was able to revert this secretory framework of the colon to water and electrolytes by reversing the hypervolemia.

## RESUMO

**Racional:**

A insuficiência renal é doença que afeta diversos órgãos por provocar hipervolemia e quadro urêmico ao organismo. A doença atinge mais de 500 milhões de pessoas em todo o mundo, e poucos estudos trazem sua influência sobre o trato gastrointestinal.

**Objetivo:**

Avaliar a influência da hipervolemia induzida pela nefrectomia 5/6 sobre a permeabilidade colônica para água e eletrólitos.

**Método:**

Foram utilizados 60 ratos machos Wistar, pesando entre 280-300 g divididos em três grupos: 3, 7 e 14 dias de pós-nefrectomia. Cada grupo foi formado por um controle e outro parcialmente nefrectomizado. Para os estudos de permeabilidade colônica, os animais foram submetidos à perfusão colônica com solução de Tyroad contendo fenolftaleína por 60 min. Diferenças entre as concentrações de Na+, K+, e Cl^-^ foram utilizadas para calcular a taxa de permeabilidade colônica para os eletrólitos e as de fenolftaleína para avaliar a taxa de secreção e absorção de água.

**Resultados:**

A secreção colônica de água e eletrólitos ocorreu de forma expressiva no grupo 7 dias pós-nefrectomia. Avaliações hemodinâmicas e bioquímicas determinaram a evolução da insuficiência renal nos três grupos e o polietilenoglicol mostrou-se eficaz na reversão da capacidade secretora do cólon.

**Conclusão:**

O quadro de hipervolemia estabelecido a partir dos sete dias pós-nefrectomia 5/6 provocou acentuada secreção colônica para água e eletrólitos. O organismo apresenta secreção colônica progressiva a medida que aumenta a volemia sanguínea; por outro lado, o polietilenoglicol foi capaz de reverter esse quadro secretor do cólon para água e eletrólitos por reverter o quadro hipervolêmico.

## INTRODUCTION

The kidneys are responsible for regulating body fluids and eliminating unwanted products from the body. Patients with renal failure do not maintain metabolic and hydroelectrolytic balance resulting in plasma hypervolemia that compromises the functioning of various systems or organs^2^.

Kidney failure is a relevant public health problem because it has epidemic proportions. The disease affects more than 500 million people worldwide^23^. In Brazil, the number of dialysis patients is around 100,000, with a hospital stay rate of 4.6% per month and mortality of 17% per year^21^. Clinical observations show that plasma hypervolemia in hemodialysis patients is associated with gastrointestinal disease. There is also a strong association between hypervolemia and gastric emptying time in patients with terminal renal failure. The results show that 68% of renal patients have dyspepsia and reduced gastric emptying time when compared to patients without this disease^5^.

Research on rats submitted to 5/6 nephrectomy has shown that there is a significant reduction in gastric emptying, and when submitted to bilateral nephrectomy they present intense intestinal secretion of water and electrolytes in the duodenal, jejunal and ileal segments^3^ ^,^ ^10^ ^,^ ^11^. Any kind of electrolyte imbalance is dangerous since the optimal functioning of the body depends on it. Low sodium (hyponatremia), for example, can cause confusion and muscle weakness^4^ ^,^ ^9^ ^,^ ^15^.

The colon is part of the intestine that suffers many influences of intrinsic factors directly compromising its absorptive and secretory capacity^15^. In this aspect, the hypervolemia caused by renal failure would alter the permeability of the colon causing dehydration and electrolyte imbalance in the body.

The aim of this study was to evaluate the colonic permeability to water and electrolytes in induced hypervolemia consequent to 5/6 nephrectomy. 

## METHOD

The project was approved by the Animal Use Ethics Commission of the Federal University of Ceará, Sobral, CE, Brazil, with protocol number 11/15. 

Male Wistar rats weighing between 280-300 g were used. The animals were kept in cages with a maximum of five at an average temperature of 24±2º C in 12 h light/dark alternation cycles, receiving standard feed (Nuvilab^®^) and water ad libitum.

Sixty animals were divided into three groups: 3, 7 and 14 days, each group consisting of a control (SHAM) and a partially nephrectomized control (PNX).

### Surgical procedures

The animals were anesthetized with xilasine (Virbaxil^®^ 2%, Virbac, 20 mg/kg) and ketamine (Sed omin, Konig do Brasil, 25 mg/kg) both intramuscularly. Initially, they underwent left lateral lumbotomy, when the upper and lower left kidney nephrectomy was performed. For this, the animals’ hair was shaved in the renal bed region and the abdominal cavity was opened. Then the left kidney was released, decapsulated with special care not to damage the adrenal gland, and both left and upper left kidney poles were removed by scalpel incision. Bleeding was stopped using an absorbable hemostatic (Surgicel^®^, Ethicon), and the surgical wound was carefully closed by suturing in two planes with 4.0 nylon thread.

After seven days, the animals were again anesthetized as previously described and submitted to right lateral lumbotomy. The right kidney was decapsulated, the renal hilum was ligated, the right kidney was removed and the surgical wound was carefully closed by suturing in two planes with 4.0 nylon thread. The end result of both operations was a partial nephrectomy at 5/6^1^.

For all experimental group, a SHAM group was submitted to the same laparotomy and renal pedicle manipulation, except for subtraction of renal mass.

During the 3^rd^, 7^th^ and 14^th^ days, a laparotomy of approximately 2 cm was performed on the SHAM and PNX animals to visualize the abdominal viscera. After isolation of the colon, polyvinyl cannulae (0.3 cm OD/0.2 cm OI) were introduced at their proximal and distal end by surgically created fistulas that were occluded by obstructive ligation with surgical threads (silk 3.0), forming in such a way between cannulae and colon segment, the circuit to be perfused. After cannula implantation, the loops were reintroduced into the abdominal cavity.

For infusion, modified Tyroad’s solution (NaCl 6.5 g/l; KCl 0.14 g/l; CaCl 0.12 g/l; NaHCO3 0.2 g/l; NaH2PO4 0.01 g/l) and phenolphthalein 50 mg/ml as a non-absorbable marker, were used. The liquid was kept warm in a water bath at 37° C and infused by a peristaltic pump (Milan BP-200) at a constant rate of 0.14 ml/min. Once the preparation was stabilized for 30 min, the infusion was collected in test tubes every 20 min for 60 min (three samples).

To determine the effect of dehydration on colonic permeability, a group of animals seven days post-nephrectomy 5/6 received 4 h before subcutaneous colonic infusion, 2.5 ml of PM 20,000 polyethylene glycol solution (30%) on each side of the animal’s back^14^.

### Hemodynamic parameters by tail plethysmography

For measurements of systolic blood pressure and heart rate during the 3^rd^, 7^th^, and 14^th^ days, SHAM and PNX rats were placed in a heated acrylic cylindrical tube and adequately ventilated for systolic blood pressure measurements. For such procedure, the tail was fitted to a rubber cuff and fitted to the proximal region of the tail and attached to the sphygmomanometer to automatically inflate and deflate at fixed intervals of approximately 50 s. The signal was captured and connected to a signal amplifier, RTBP 2000 Rat Tail Blood Pressure System For Rats and Mice (Kent Scientific Corporation) and connected to a PowerLab/400 digital analog converter (ADInstruments, Australia).

The animals were kept for a period of adaptation and stabilization of the signals; thereafter, the 30-min experiments were started, being the first 10 min to adequate, and the systolic blood pressure and heart rate were simultaneously checked for the next 20 min.

### Central venous pressure by jugular cannulation

To measure central venous pressure, before colonic perfusion, animals were anesthetized with 1.2 mg/kg of urethane, and had the right jugular vessel cannulated using cannulas (PE50); the cannulas were filled with saline and heparin (500 UL/ml).

At the time of the experiments, the cannulas were previously inserted into the vessels and coupled to a PowerLab/400 biological signal acquisition system (AD Instruments, Australia) to obtain continuous hemodynamic records, which were stored in a computer.

### Blood volume

Blood volume was determined after colonic perfusion by hemodilution technique. For this, the animals received intravenous injection (0.2 ml) of Evans blue solution (40 mg%) via the right jugular vein. At the time of sacrifice, blood was collected by cardiac puncture and centrifuged at 2,800 rpm for 20 min. Evans blue concentration was determined by spectrophotometry (620 nm). Blood volume was determined from hematocrit and plasma volume^8^. 

### Biochemical parameters

Phenolphthalein concentration was determined by spectrophotometry. Differences between concentrations were employed to evaluate the secretion/water absorption rate by the infused segment^12^.

Sodium, potassium and chloride concentrations were measured by the AVL Roche^®^ 9180 selective ion. Differences between their concentration values ​​were used to calculate the electrolyte colon transport rate. Standard colorimetric tests (Labtest) were used to determine urea and creatinine values.

### Statistical analysis

Data on hemodynamic and biochemical parameters (urea and creatinine) obtained from each of the groups were studied in the mean **±** E.P.M. Student’s t-test followed by ANOVA was used to evaluate statistical differences between groups and different experimental protocols. P values ​​<0.05 were considered significant.

## RESULTS

**FIGURE 1:**
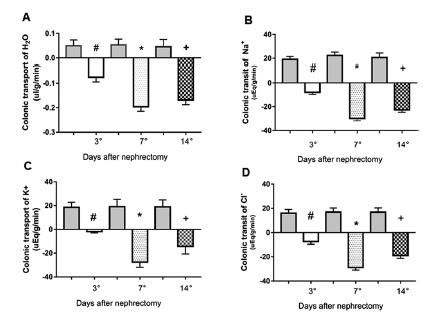
Study of colonic permeability of water and electrolytes in sham-operated and partially nephrectomized (PNX) rats for 3, 7 and 14 days after nephrectomy 5/6: A) absorption and or colonic secretion of water; B) absorption and or colonic secretion of sodium Na^+^; C) absorption and or colonic secretion of potassium K^+^; D) absorption and or colonic secretion of chlorine. Negative bars represent the mean colonic secretion values while positive verticals represent the mean absorption values and the vertical lines indicate the standard error of the mean, *p<0.05. Fifteen animals were used to compose the SHAM control group and fifteen for the NPX group.

Colonic water absorption and or secretion rates revealed very equidistant absorption levels in the SHAM groups (3, 7 and 14 days, 0.049±0.009 ul/g/min, 0.050±0.008 ul/g/min, 0.011±0.003 ul/g/min, respectively, p <0.05) and variable secretion in the three groups 3, 7 and 14 days PNX (-0.07±0.007ul/g/min, -0.18±0.01ul/g/min, -0.16±0.01 ul/g/min, respectively #*+p<0.05); however, the partially nephrectomized 7-day group had the highest colonic H_2_O secretion (Figure 1A).

Sodium in the SHAM groups (3, 7 and 14 days) had similar absorption (19.08±1.09 uEq/g/min, 22.38±1.42 uEq/g/min, 23.73±1,68 uEq/g/min respectively, p<0.05) and secretion in groups 3, 7 and 14 days PNX (-8.27±0.65 uEq/g/min, -28.42±1.60 uEq/g/min, -24.85±1.61 uEq/g/min, respectively, #*+p<0.05); however, the partially nephrectomized seven-day group of animals had higher colonic Na^+^ secretion than the following 3 and 14-day groups (Figure 1B).

Potassium in the SHAM groups (3, 7 and 14 days) had similar absorption (18.96±1.63 uEq/g/min, 17.76±1.50 uEq/g/min, 16.9±1.965 uEq/g/min respectively, p<0.05). Groups 3, 7 and 14 days PNX presented the following secretions: -1.8±0.46 uEq/g/min, -24.01±1.44 uEq/g/min, -17.24±1,81 uEq/g/min, α #*p<0.05) in partially nephrectomized 7-day animals there was marked K^+^ secretion (Figure 1C).

Chlorine in the SHAM groups (3, 7 and 14 days) was absorbed at 16.04±1.37 uEq/g/min, 16.88±1.4 uEq/g/min, 17.33±1.58 uEq/g/min respectively, α#*p<0.05 and high secretion in groups 3, 7 and 14 days PNX (-9.42±1.03 uEq/g/min, -27.49±1.86 uEq/g/min, -18.23±1.71 uEq/g/min, respectively, α#*p<0.05); however, animals with seven days PNX had higher Cl^-^ secretion compared to groups, 3 and 14 days (Figure 1D).

**FIGURE 2:**
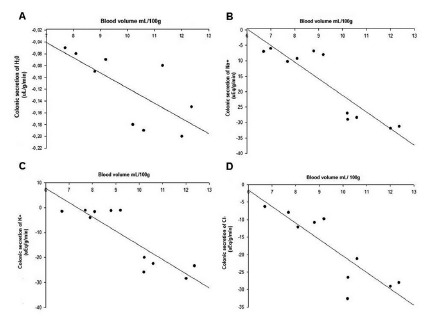
Relationship between the volume of 5/6 nephrectomized rats and colonic secretion during the three and seven days after 5/6 nephrectomy: A) linear regression curve between blood volume and colonic water secretion (H_2_O) *p<0.05; B) linear regression curve between blood volume and colonic sodium secretion (Na^+^) *p<0.05; C) linear regression curve between blood volume and colonic potassium secretion (K^+^) *p<0.05; D) linear regression curve between blood volume and colonic chlorine secretion (Cl^-^) *p <0.05

Colonic water secretion in nephrectomized animals was proportional to blood volume and can be represented by linear regression curve established with the following equation Y= -0.02X + 0.13, where Y represents the secretion rate and X is the blood volume (r=0.71, Figure 2A).

Sodium secretion established an even closer correlation with blood volume than water, forming a linear regression curve established with the following equation Y= -5.19X + 31.3, Y represents secretion rate and X volume blood pressure (r=0.86, Figure 2B).

Potassium strongly correlated blood volume with a linear regression curve established by the following equation Y= -5.69X +41.85, where Y represents the rate of secretion, X blood volume (r=0.87, Figure 2C )

Chlorine, similar to other electrolytes, also had a strong correlation between its secretion and blood hypervolemia and can be represented by a linear regression curve established with the following equation Y= -4.70X + 26.6, where Y represents secretion rate, X blood volume (r= 0.85, Figure 2D).

Hemodynamic variations - systolic blood pressure, heart rate, central venous pressure and blood volume - were significant in animals 7 and 14 days after nephrectomy; differences between three-day PNX animals and SHAM animals (128.7±3.3 mmHg; 369.9±3.4 bpm; 1.2±0.13 cm H_2_O; 7.067±0.35 ml; respectively*, 116.1±1.7 mmHg; 370±2.9 bpm; 1.7±0.8 cm H_2_O; 6.22±0.38 ml; respectively,* p<0.05) did not express relevance (Table 1).

**TABLE 1:** Hemodynamic evaluation of SHAM groups and 3.7 and 14 days partially nephrectomized.

Variable	SHAM	3 Days	7 Days	14 Days
Blood presure (mmHg)	116.1 ± 1.7	128.7 ± 3.3	137.4 ± 3.2*	149.6 ± 2.1
HB (beats/min)	370 ± 2.9	369.9 ± 3.4	383.5 ± 3.3	388.8 ± 1.4
CVP (cmH_2_O)	1.7 ± 0.8	1.2 ± 0.13	3.5 ± 0.17	4.1 ± 0.19
Blood volume (ml/100g)	6.22 ± 0.8	7.067 ± 0.35	12.14 ± 0.37*	14.83 ± 0.82

Blood volume increase in animals with seven days PNX compared to animals with three PNX (7.067±0.35 ml/100 g) and (12.14±0.37 ml/100 g) was significant, the same can be observed in relation to blood pressure (128.7±3.3 mmHg) and (137.4±3.2 mmHg), respectively.

**FIGURE 3:**
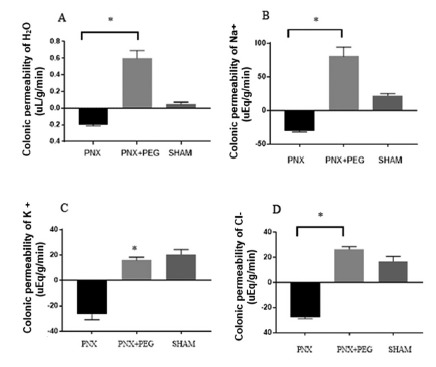
In the group seven partially nephrectomized (PNX), partially nephrectomized days treated with polyethylene glycol (PNX + PEG) and SHAM: A) colonic absorption and H2O secretion; B) colonic absorption and Na^+^ secretion; C) colonic absorption and K^+^ secretion; D) colonic absorption and Cl^-^ secretion. Negative vertical bars represent mean values of colonic secretion; positive vertical bars represent mean absorption values; and vertical lines indicate the standard error of the mean. *p<0.05; n=5 to 8 animals per group.

Polyethylene glycol promoted porous retraction of blood volume by dehydration in partially nephrectomized seven-day animals. It was able to reverse water secretory capacity (-0.18±0.01 ul/g/min vs. 0.70±0.09 ul/g/min), respectively; *p<0.05) and sodium (-28.42±1.60 uEq/g/min vs. 0.53±6.35 uEq/g/min respectively; *p<0.05) by nephrectomy-induced colon 5/6 (Figure 3).

Plasma urea levels among nephrectomized animals were elevated in groups 7 and 14 days PNX 114.8±1.8 mg/dl and 165.6±7.2 mg/dl respectively, *p<0.05. The 3-day PNX group (79.8±3.63 mg/dl) did not differ significantly from SHAM (79.8±3.63 mg/dl) *p<0.05.

As for plasma creatinine level, a small evolution was observed among nephrectomized animals. The 14 days PNX group presented the highest creatinine level (1.3±0.07, *p<0.05), followed by the seven days PNX group (0.95±0.02, *p<0 .05). The three-day PNX group (0.45±0.08 mg/dl) was not very different from the SHAM (0.30±0.07 mg/dl, *p<0.05, Table 2).

**TABLE 2:** Plasma biochemical analysis of urea and creatinine from SHAM control and experimental groups PNX 3, 7 and 14 days after nephrectomy 5/6

Biochemical variables	SHAM	3 days	7 days	14 days
Urea (mg/dl)	55.6 ± 1.07	79.8 ± 3.63	114.8 ± 1.8	165.6 ± 7.2*
Creatinine (mg/dl)	0.30 ± 0.07	0.45 ± 0.08	0.95 ± 0.02	1.3 ± 0.07*

## DISCUSSION

In this study it was shown that there is colonic secretion of water and electrolytes during the 3, 7 and 14 days after nephrectomy 5/6, and the most significant results were found in the group 7 days followed by 14. During the three days after nephrectomy no significant results were found for water and electrolyte secretion when compared to the SHAM group.

The relationship between hypervolemia on colonic water secretion in partially nephrectomized animals can be confirmed by the linear regression curve established in Figure 2. This secretagogue effect of colon to water is due to two very important factors. The first is because PNA inactivates renin and aldosterone, two potent hormones that act on the colon by stimulating water absorption^17^ ^,^ ^19^. The second is that water absorption by the colon occurs by the osmotic gradient created by the high concentrations of ions in space. intercellular. Most of this osmosis occurs through the closed joints between the apical edges of the epithelial cells. However, a smaller proportion occurs through the intestinal cells themselves. If ion concentrations are high in the colonic lumen - a fact observed when the colon becomes an electrolyte secretor - the colon excretes water obeying the direction of the osmotic gradient^6^.

The seven days group presented higher electrolyte secretion, Na^+^ (- 28.42±1.60) followed by Cl^-^ (-27.49±1.86), with very equidistant results. Figures 2B and 2D show a strong relationship between hypervolemia and Na^+^ and Cl^-^ secretion through a linear regression curve. The significant loss of these electrolytes by the colon is due to changes in the activity of colonic mucosal ATPases, as they are responsible for the electrochemical differential of the intestinal mucosa that promotes sodium absorption and consequently chlorine absorption.

The proximity in the secretion values ​​of these electrolytes is due to their behavior similar to the absorptive pattern by the colon, the result of a chemical and electrical potential generated from sodium itself and occasionally by other electrolytes with the aid of the ATPase protein found in the basolateral membrane brush edges that promote the absorption of these ions. Meanwhile, potassium ions are transported in the opposite direction in exchange for sodium ions^6^ ^,^ ^13^.

Increasing blood volume triggers colonic secretion to water and electrolytes because it promotes the release of natriuretic peptides, a group of hormones secreted by different tissues, especially the heart. These have their role in various organs, but there is emphasis on the kidney, by increasing the excretion of water and sodium through inhibition of NA^+^/K^+^ ATPase pump and inhibition of renin and aldosterone^17^ ^,^ ^19^.

There are at least three peptides related to natriuresis in the intestines and colon: STa, Gn and UGn. They bind to a receptor present on the brush border surface of the intestine and colon. These receptors were identified as a member of guanylate cyclase and were designated GC-C. Its activation increases intracellular levels of cGMP, which induces an intracellular cascade that culminates in the activation of protein kinase GII (PG and PKA), leading to activation of the cystic fibrosis transmembrane regulatory channel (CFTR), which promotes increased secretion of chlorine, bicarbonate and water in the intestine^20^ ^,^ ^22^.

Polyethylene glycol is commonly used for experimentally reducing plasma volume, causing acute dehydration in cells. In studies of gastric emptying in PNX animals, it was able to reverse gastric emptying reducing hypervolemia^11^. In this study, it was able to reverse colonic secretion of water and sodium by returning its absorptive capacity in partially nephrectomized animals (Figure 3).

Acute retraction of blood volume can be achieved by dehydration, reduced sodium intake or bleeding and results in the maximization of fluid and electrolyte absorption through the intestinal epithelium. This occurs by activating cardiovascular receptors, which trigger neural flexion, leading to the release of norepinephrine by the splanchnic and mesenteric nerves^18^.

Therefore hypervolemia would be the main cause of this colon secretagogue phenomenon observed in this study. Many researchers, through clinical and experimental observations, indicate that the gastrointestinal tract may modify its absorptive pattern, making it even secretory when subjected to acute plasma volume variations^3^ ^,^ ^10^ ^,^ ^16^ ^,^ ^17^ ^,^ ^23^.

The high blood pressure in groups 7 and 14 days PNX compared to group 3 days PNX is due to the volume overload that occurred during this period. Blood volume in groups 7 and 14 days (11.12±0.37 ml; 15.83±0.82 ml, respectively, p<0.05) was also quite pronounced when compared to the group three days ( 8.067 ml±0.35).

Plasma electrolyte concentrations, Na^+^, K^+^ and Cl^-^ (141.1±1.5 mEq/ml, 7.1±0.5 mEq/ml and 99.2±1.0 mEq/ml, respectively) at seven PNX days were higher than those at three PNX days (138.0±1.7 mEq/ml, 3.8±0.1 mEq/ml and 102.5±1.2 mEq/ml, respectively; p< 0.05). This explains the expansion of blood volume during the period of renal failure. After 70% loss of renal mass, the body accumulates salt and water over time, which causes hypertension due to plasma volume overload^6^. Volume overload also increased central venous pressure between groups 7 and 14 days PNX compared to three days PNX (7.1±0.19 cm H_2_O, 5.2±0.17 cm H_2_O vs. 2,2±0.13 cm H_2_O) respectively, p<0.05.

The progressive increase in urea and creatinine levels in groups 3, 7 and 14 days PNX (79.8±3.63, 114.8±1.8, 165.6±7.2, respectively, p<0.05) and (0.30±0.07, 0.45±0.08, 0.95±0.02, 1.3±0.07 respectively, p<0.05) were the basis for determining the evolution of renal failure in rats submitted to nephrectomy 5/6. Uremic toxins generated in renal dysfunction are responsible for the progression of chronic kidney disease by inducing loss of residual renal function^7^. The mechanisms of colonic secretion and absorption for water and electrolytes in the hypervolemic situation should be better studied in humans since renal patients are at risk of an electrolyte imbalance and its manifestation is very gradual, most often asymptomatic and may be fatal.

## CONCLUSION

The hypervolemia established in the rats from the 7^th^ day after 5/6 nephrectomy caused a marked colonic secretion to water and electrolytes. The body presents progressive colonic secretion as blood volume increases; on the other hand, polyethylene glycol was able to revert this colonic secretory picture to water and electrolytes by reversing the hypervolemia.
